# Comparison of the Effects of Lidocaine Versus Magnesium Infusion on Quality of Recovery After Nasal Bone Fracture Surgery

**DOI:** 10.3390/medicina60121939

**Published:** 2024-11-25

**Authors:** Eun Kyung Choi, Jongyoon Baek, Kyu Jin Chung

**Affiliations:** 1 Department of Anesthesiology and Pain Medicine, Yeungnam University College of Medicine, 170, Hyeonchung-ro, Nam-gu, Daegu 42415, Republic of Korea; zzini0527@naver.com (E.K.C.); jongyoonbaek@gmail.com (J.B.); 2 Department of Plastic and Reconstructive Surgery, Yeungnam University College of Medicine, 170, Hyeonchung-ro, Nam-gu, Daegu 42415, Republic of Korea

**Keywords:** lidocaine, magnesium, quality of recovery-40 questionnaire

## Abstract

*Background and Objectives*: Postoperative recovery from general anesthesia is a multidimensional process, and patient-centered outcome assessment should be considered an important indicator of recovery quality. This study compared the effectiveness of intraoperative lidocaine and magnesium on postoperative recovery in nasal bone fracture surgery, using the quality of recovery-40 questionnaire (QoR-40) to assess recovery quality and pain intensity. *Materials and Methods*: A total of 74 patients scheduled for elective closed reduction surgery for isolated nasal bone fracture were assigned to the intraoperative infusions of lidocaine or magnesium. Immediately after anesthetic induction, 1.5 mg/kg lidocaine or 20 mg/kg magnesium was administered over 10 min, followed by a continuous infusion of 2 mg/kg/h lidocaine or 20 mg/kg/h magnesium until the end of the surgery. The primary outcome variable was the QoR-40 survey on postoperative day 1. The secondary outcome variables included postoperative pain intensity, sedation score, the incidence of postoperative nausea and vomiting, and other side effects. *Results*: The global QoR-40 score at postoperatively 24 h was comparable between the intraoperative lidocaine and magnesium groups. Postoperative pain 30 min after surgery was significantly lower in the lidocaine group than in the magnesium group (*p* = 0.01), along with lower rescue analgesic consumption (*p* = 0.003), but pain intensity was not different at other time points (2, 6, and 24 h). The incidence of nausea and vomiting and the sedation score in the post-anesthetic care unit were not significantly different between the two groups. *Conclusions*: Intraoperative lidocaine and magnesium had no difference in the scores of postoperative QoR-40, but lidocaine was associated with lower postoperative pain scores and analgesic requirement in comparison to magnesium in the closed reduction of nasal bone fractures.

## 1. Introduction

Postoperative recovery from general anesthesia is a multidimensional process that encompasses the pain, cognition, emotion, and functional domains. Not only the restitution of physiologic parameters, but also patient-centered outcome assessments, such as comprehensive satisfaction, should be focused on as important indicators of the quality of postoperative recovery.

Lidocaine or magnesium has been used as an anesthetic adjuvant in an attempt to attenuate postoperative pain [1,2]. Both drugs have the properties of NMDA antagonists, which can affect the perception of pain. Many previous studies have demonstrated the antinociceptive effect of both drugs with better analgesia and lower opioid consumption. In addition, unlike opioids, they have fewer side effects [3,4]. In the extended context of postoperative pain control, the assessment of the quality of recovery with perioperative systemic lidocaine or magnesium has been studied in various subsets of the surgical field [5,6], and the quality of recovery-40 questionnaire (QoR-40) has been commonly used to evaluate the early quality of recovery. QoR-40, a patient-reported outcome measurement scale, has high validity and reliability in patients undergoing general surgery [7,8].

Nasal bone fracture is the most common fracture of the face, accounting for about 40% of all facial bone fractures, and closed reduction is the standard surgical treatment [9]. By the epidemiological studies for nasal bone fracture, males aged 10 to 40 years old had the highest rate of incidence [9]. Closed reduction for isolated nasal bone fracture needs nasal packing to prevent postoperative bleeding, which can lead to patient discomfort and airway problems besides acute surgical pain [10]. In addition, because airway-related complications after tracheal extubation including the residual effects of hypnotics or muscle relaxants, laryngeal edema, and risk of aspiration from nasal bleeding are considered risky, awake extubation can be preferred and the resulting emergence agitation may be intensified [11,12]. Therefore, the perioperative use of lidocaine or magnesium may decrease postoperative pain intensity and stress response such as emergence agitation, and subsequently improve the quality of recovery after nasal bone surgery.

This study aimed to compare the effects of intraoperative systemic lidocaine and magnesium on the quality of recovery by assessing the QoR-40 in patients undergoing nasal bone fracture surgery. Furthermore, we evaluated the postoperative pain intensity, analgesic requirement, sedation score, and incidence of postoperative nausea and vomiting after nasal surgery.

## 2. Materials and Methods

### 2.1. Study Design/Sample

To address the research objective, we designed a prospective randomized controlled trial. This study was approved by the Institutional Review Board (YUMC 2018-09-035) and registered at the Clinical Research Information Service (KCT0008804). Written informed consent was obtained from all the patients. We included patients scheduled for elective closed reduction surgery for isolated nasal bone fracture under general anesthesia, aged 20–65 years, with American Society of Anesthesiologists (ASA) grade I or II. The exclusion criteria included psychological disorders, chronic analgesic use, allergies to experimental drugs, clinically significant cardiovascular or kidney or liver disease, heart blocks greater than the first block, uncontrolled hypertension, or a body mass index > 30 kg/m^2^.

### 2.2. Variables

The primary predictor variables were the intraoperative infusions of either lidocaine or magnesium. The patients were randomly allocated to the lidocaine group (group L) or magnesium group (group M) using a computer-generated random number table and the assignments were placed in a sealed envelope. All the steps involved in the study (patient assignment, study drugs, and outcome assessment) were conducted in a double-blind fashion.

The primary outcome variable was the QoR-40 survey on postoperative day (POD) 1. The secondary outcome variables were postoperative pain intensity, sedation score, the incidence of postoperative nausea and vomiting, and other side effects such as mouth numbness and atrioventricular block. In the post-anesthetic care unit (PACU) and ward, postoperative pain intensity was checked at 30 min and 2, 6, and 24 h using a numeric rating scale (NRS: 0 = no pain; 10 = the worst pain). If the NRS score > 5, or the patient requests the analgesics, intravenous fentanyl 50 mcg was given, and the incidence and dose of analgesics requirement were recorded. The QoR-40 survey was conducted on POD1, 6–8 PM. The preoperative QoR-40 was also assessed on the day before surgery. QoR-40 consists of 40 questions on physical independence, psychological support, physical comfort, emotional state, and pain (total score: 40–200) [13]. A higher score indicated an excellent quality of recovery. The survey was conducted in the presence of a researcher and took about 5–10 min. In the PACU, the sedation score was analyzed using a 5-point scale [14]: (1) fully conscious; (2) slightly drowsy; (3) seemingly sleeping but immediately reacting to verbal stimulation; (4) seemingly sleeping but slowly reacting to verbal stimulation; and (5) seemingly sleeping but reacting to shaking or pain, not verbal stimulation. Other adverse symptoms such as nausea, vomiting, mouth numbness, and atrioventricular block were also evaluated.

Covariates included patients’ demographics such as age, sex, height, weight, ASA grade, duration of anesthesia, and preoperative scores of QoR-40, comprising physical independence, psychological support, physical comfort, emotional state, and pain sections.

### 2.3. Surgical Technique: Closed Reduction

The patient is positioned supine with neck extension on the operating table. Following general anesthesia, Chlorhexidine is used to sterilize the face and nasal cavity. Gauze soaked in a mixture of 2% lidocaine and 1:100,000 epinephrine is inserted into the superior and middle meatus. After 10 min, the nasal packing gauze is removed, and the nose is palpated to identify depressed areas using the left thumb and index finger. Nasal elevators are then employed to assess the displacement of nasal bone segments and perform nasal bone reduction. Merocel^®^ packing (Medtronic Inc., Minneapolis, MN, USA) is placed in both superior meatus, followed by saline infiltration. An external splint or tape is often placed over the nasal dorsum following the reduction.

### 2.4. Data Collection Methods

After routine monitoring, propofol, remifentanil, and rocuronium were administered without premedication to induce anesthesia, followed by endotracheal intubation. Sevoflurane in 50% oxygen/air and remifentanil was used for anesthetic maintenance; 1.5 mg/kg lidocaine or 20 mg/kg magnesium was administered over 10 min and continuous infusion was performed at 2 mg/kg/h lidocaine or 20 mg/kg/h magnesium until the end of the surgery. After the surgery, the residual neuromuscular blockade was reversed, and extubation was performed when the patient had regular breathing.

### 2.5. Data Analyses

Based on a previous study, the difference in the total QoR-40 score postoperatively (primary outcome) between the experimental groups was more than 10 points, which was considered significant for improving recovery quality [15]. Therefore, the sample size was calculated with a standard deviation of 13 [15] to achieve a power of 0.9 and an α of 0.05 (effect size = 0.769). Allowing the dropout rate of 10%, 82 patients per group was required. Data were expressed as mean ± SD or number (%) or median with interquartile range. A statistical analysis was performed using the SPSS software (version 23.0, IBM Corp., Armonk, NY, USA) and a one-way analysis of variance (ANOVA) test, Kruskal–Wallis test, chi-squared test, or Fisher’s exact test was used as appropriate. Statistical significance was set at *p* < 0.05.

## 3. Results

Among the eighty-two patients, eight patients were excluded for the following reasons: the surgical plan was changed in two patients, and six patients declined the postoperative follow-up QoR-40. Both groups had equal numbers. The mean ages for the lidocaine and magnesium groups were 37.2 ± 11.8 and 36.4 ± 14.1 years, respectively (*p* = 0.790). Male–female ratios were 1.06 and 1.47, respectively (*p* = 0.640). Neither group showed significant differences in the patient characteristics or baseline data (Table 1). The total scores of QoR-40 were comparable between the lidocaine and magnesium infusion groups. The pre- and postoperative sub-scores of each dimension (physical independence, psychological support, physical comfort, emotional state, and pain) were not significantly different between the two groups. The change in the total QoR-40 scores at 24 h postoperatively compared to the preoperative scores did not show a significant difference between the two groups (Table 2). Postoperative pain 30 min after surgery was significantly lower in the lidocaine administration group than in the magnesium group (*p* = 0.010); however, pain intensity was not different at other time points (2, 6, and 24 h). The consumption of rescue analgesics was lower in the lidocaine group than in the magnesium group (*p* = 0.003) (Table 3). There were no significant differences in the other recovery profiles, including nausea, vomiting, mouth numbness, and atrioventricular block, during the first 24 h postoperatively, and the sedation score in each group and the length of PACU stay were comparable upon arrival and discharge from the PACU. The length of stay in the PACU did not differ between the groups (Table 4).

## 4. Discussion

The QoR-40 scores of the lidocaine and magnesium infusion groups were comparable in patients undergoing nasal bone fracture surgery. However, the postoperative analgesic effect of lidocaine was superior to that of magnesium. No adverse effects were associated with the use of lidocaine or magnesium at the doses used in this study.

Improving the quality of recovery, reducing postoperative complications, and facilitating early discharge are essential in anesthesia and surgery. Therefore, in addition to postoperative physiological parameters such as pain, nausea, vomiting, and shivering, patient-centered outcomes regarding comprehensive recovery quality are important in the assessment of early postoperative health status. QoR-40 is an effective index for assessing a patient’s perception of recovery after general anesthesia [7]. Previous studies have evaluated early recovery quality using the QoR-40 in various surgical settings [2,15]. This 40-item questionnaire is composed of five clinically relevant dimensions, which is a simple and convenient measurement of postoperative recovery quality. Myles et al. showed good validity and reliability of QoR-40 in surgical practice [7]. Therefore, we used the QoR-40 questionnaire to compare the effects of lidocaine and magnesium on the postoperative recovery quality. Based on previous studies, there have been some reports showing a difference in the quality of recovery with the use of lidocaine or magnesium during various surgical procedures. However, there has not been a report describing patients who underwent nasal bone fracture surgery.

Lidocaine, a local anesthetic, has been shown to have a postoperative analgesic effect with reduced opioid consumption in some cases [2]. The possible mechanisms to explain this might be multifactorial: the prevention of central hyperalgesia by blocking the sodium channels of afferent pain fibers, anti-inflammatory properties, and blocking NMDA receptors [2]. In addition, non-analgesic benefits such as improved gastrointestinal motility and reduced nausea and vomiting have been reported [16]. These analgesic and non-analgesic properties may contribute to improved postoperative recovery quality after surgery. Kim et al. reported that intraoperative lidocaine administration led to improved quality of recovery, as measured by the QoR-40, in patients undergoing thyroid surgery [5]. However, contradictory results were observed [17].

Magnesium sulfate, as an analgesic adjunct, has been widely studied, supporting its benefits in reducing postoperative pain, rescue analgesia, and anesthetic drug requirements [18,19]. It modulates central sensitization by blocking NMDA receptors in a voltage-dependent manner and inhibits the catecholamine release [20]. Another consideration is that magnesium sulfate acts as a calcium channel blocker, which supports more standard therapy as other adjuvant effects (myocardial protection, antiarrhythmic action, and the prevention of vasospasm) [21]. Do et al. reported that the antiemetic effect of magnesium has also been attributed to a reduction in perioperative opioid consumption [22]. Therefore, magnesium has been used as part of a multidrug anesthetic plan and has also been studied from the perspective of the quality of recovery. A report by Lu et al. showed that magnesium administration improved functional recovery after laparoscopic cholecystectomy [17].

In previous studies regarding both drugs on the postoperative recovery quality, Kim et al. showed that intraoperative lidocaine infusion improved QoR-40 scores with emotional state and pain scores after breast cancer surgery compared to a control group [6]. In contrast, in a study by Lu et al., magnesium led to better QoR-15 scores by improving physical independence and comfort in patients who underwent laparoscopic cholecystectomy, whereas lidocaine was insufficient to induce QoR improvement [17]. In this study, the total QoR-40 and sub-dimensional QoR scores were comparable between the lidocaine and magnesium groups. Here, we did not show the effect of lidocaine or magnesium alone on recovery quality. Therefore, subsequent studies with a control group would be beneficial to establish and clarify the baseline nature of each drug on the comprehensive recovery quality in patients undergoing nasal bone fracture surgery. In addition, we compared the analgesic effects of lidocaine and magnesium on postoperative pain for 24 h in terms of non-opioid perioperative analgesic adjuvants. Postoperative pain control is an important factor in determining a patient’s quality of recovery. In this study, lidocaine infusion had a better analgesic effect 30 min postoperatively and reduced the total rescue analgesic requirements. This result is consistent with some reports in which systemic lidocaine was shown to lower morphine consumption compared to magnesium [1]. There have been contrary results [23], and the discrepancy might be associated with surgery type or drug administration timing.

This study has some limitations. First, the dose of each study medication might not be equipotent to compare the quality of recovery in the closed reduction of nasal bone fractures although lidocaine or magnesium sulfate was administered as a commonly used dose based on previous studies. Second, it is likely that the total dose of lidocaine or magnesium sulfate may be inferior to clinical importance due to this surgical setting being a relatively short-term surgery. So, further studies on dose titration and the optimal dose of both drugs are required considering the above limitations. Third, the sample size calculation was based on a difference of 10 or more of the QoR-40. However, the sample size may be underpowered to check the quality of recovery (the mean difference in the two groups was just 4.81 at POD1). Finally, though QoR-40 would be a useful tool to evaluate on quality of recovery in perioperative clinical studies, other assessment methods or objective outcomes may be needed for the identification of individual patient recovery.

## 5. Conclusions

In conclusion, the effects of magnesium and lidocaine on the improvement of functional recovery were comparable in patients who underwent nasal bone fracture surgery. However, the intraoperative systemic administration of lidocaine led to better postoperative pain control with reduced analgesic requirements compared with the use of magnesium in the closed reduction of nasal bone fractures. Further research with other reliable assessment tools on the quality of recovery or a higher infusion dose of lidocaine and magnesium would be needed.

## Figures and Tables

**Table 1:** Demographic and baseline characteristics.

	Group L (n = 37)	Group M (n = 37)	*p*-Value
Age (yr)	37.2 ± 11.8	36.4 ± 14.1	0.790
Sex (men/women)	19/18	22/15	0.640
Height (cm)	167.0 ± 7.9	168.0 ± 8.3	0.613
Weight (kg)	65.8 ± 12.1	66.1 ± 12.0	0.920
ASA (I/II)	28/9	24/13	0.446
Duration of anesthesia (min)	36.2 ± 6.05	37.5 ± 6.02	0.359

Values are presented as mean ± standard deviation or number. Group L, lidocaine; group M, magnesium.

**Table 2:** QoR-40 scores on the preoperative and postoperative 24 h.

QoR-40 Dimensions	Group L (n = 37)	Group M (n = 37)	*p*-Value
Physical independence			
Preoperative	23.81 ± 2.01	22.86 ± 4.23	0.225
POD1	21.02 ± 4.79	20.00 ± 5.56	0.398
Psychological support			
Preoperative	33.10 ± 2.75	32.37 ± 4.83	0.429
POD1	32.13 ± 4.21	30.94 ± 4.86	0.265
Physical comfort			
Preoperative	53.59 ± 5.26	52.86 ± 7.63	0.634
POD1	50.97 ± 8.33	50.08 ± 6.71	0.614
Emotional state			
Preoperative	37.08 ± 7.18	38.16 ± 6.22	0.492
POD1	36.48 ± 9.13	36.40 ± 8.79	0.969
Pain			
Preoperative	29.24 ± 5.75	30.86 ± 4.24	0.172
POD1	28.97 ± 6.30	27.35 ± 7.93	0.334
Total QoR-40 scores			
Preoperative	176.83 ± 17.44	177.1 ± 23.78	0.951
POD1	169.59 ± 27.62	164.78 ± 27.83	0.458
Changes in total QoR-40 score from preoperative to POD1	−12.35 ± 33.59	−7.24 ± 21.38	0.438

Values are presented as mean ± standard deviation. Group L, lidocaine; group M, magnesium; QoR-40, quality of recovery-40; POD1, postoperative day 1.

**Table 3:** Postoperative pain and rescue analgesics during the first 24 h after surgery.

	Group L (n = 37)	Group M (n = 37)	*p*-Value
Pain score			
30 min	4.02 ± 2.25	5.24 ± 1.67	0.010 *
2 h	2.86 ± 1.79	3.62 ± 2.07	0.098
6 h	2.16 ± 1.42	2.40 ± 1.81	0.524
24 h	1.24 ± 0.89	1.35 ± 1.65	0.728
Rescue analgesics, n (%)	18 (48.6)	31 (83.8)	0.003 *

* statistically significant at *p*-value < 0.05. Values are presented as mean ± standard deviation or number (%). Group L, lidocaine; group M, magnesium.

**Table 4:** Recovery profiles during postoperative 24 h.

	Group L (n = 37)	Group M (n = 37)	*p*-Value
Nausea	5 (13.5)	9 (24.3)	0.374
Vomiting	0 (0.0)	1 (2.7)	1.000
Mouth numbness	0	0	1.000
Atrioventricular block	0	0	1.000
Sedation score on PACU arrival	2 [2]	2 [2]	0.619
Sedation score on PACU discharge	1 [1, 2]	1 [1, 2]	0.708
Duration of PACU stay (min)	28.37 ± 5.65	30.27 ± 3.71	0.930

Values are presented as number (%), mean ± standard deviation, and median [IQR]. Group L, lidocaine; group M, magnesium.

## Data Availability

The data presented in this study are available upon request from the corresponding author.
